# Decision Making and Fibromyalgia: A Systematic Review

**DOI:** 10.3390/brainsci12111452

**Published:** 2022-10-27

**Authors:** Federica Alfeo, Diletta Decarolis, Livio Clemente, Marianna Delussi, Marina de Tommaso, Antonietta Curci, Tiziana Lanciano

**Affiliations:** 1 Department of Education, Psychology, Communication, University of Bari ‘Aldo Moro’, 70121 Bari, Italy; 2 Department of Basic Medical Science, Neuroscience and Sense Organs, University of Bari ‘Aldo Moro’, 70121 Bari, Italy

**Keywords:** decision making, fibromyalgia, review, chronic pain, executive functions

## Abstract

Decision making (DM) is the ability to choose among multiple options, considering external and internal variables and identifying potential paths of action that need to be assessed. Some brain areas involved in decision making are also implicated in pain processing, such as in fibromyalgia (FM). FM is a syndrome characterized by chronic widespread musculoskeletal pain and cognitive difficulties. We conducted a systematic review with the aim of identifying articles that evaluated DM in people with fibromyalgia, highlighting the main assessment tools. This work was conducted according to the PRISMA statement by consulting six online databases and providing a quality assessment of each search that met the inclusion criteria. In line with the limited interest in this in the scientific landscape to date, we found nine studies that evaluated the performance of DM in patients with FM; furthermore, we discovered that only certain types of DM were tested. The importance of our work lies in shedding light on a cognitive ability that is often undervalued in the scientific landscape but essential in everyday life. This review can serve as a starting point for further studies to clarify the relationship between DM and FM, improving understanding of the topic.

## 1. Introduction

Fibromyalgia (FM) is a chronic pain syndrome with involvement of the peripheral and central nervous systems [1]. It is characterized by chronic (more than 3 months) widespread musculoskeletal pain and several associated symptoms, such as fatigue, sleep disturbances, and other cognitive and somatic symptoms that can be debilitating and challenging [2,3]. FM mainly involves women and has an estimated prevalence in the general population of between 0.2 and 6.6%, reaching 11.4% in urban areas [4]. The word “fibrofog” was appositely coined to refer to the cognitive dysfunction associated with fibromyalgia, although there is no widely accepted definition of this term [5]. Also known as “fibromyalgia fog” and “brain fog,” the concept is used to describe the subjective experience of fog-like feelings, including a range of cognitive difficulties experienced and reported by patients with FM [5,6]. Fibrofog is a multifaceted clinical difficulty in FM that must be assessed to develop treatments that account for the importance of addressing multiple types of difficulties [7]. The mediating factors of these cognitive deficits may be related to emotional–affective problems, which are, generally, and in terms of age distribution, more relevant in patients than in healthy controls [8]. Fatigue and insomnia may also be relevant with respect to the genesis of such difficulties, although the main mediating factor is the severity of clinical pain [9]. Mechanisms related to chronic pain caused by FM can capture part of patients’ abilities [10,11,12], including decision-making skills.

Decision making (DM) is a crucial skill with a central role in daily life concerning the deliberate process of evaluating alternatives and choosing the most adaptive option to achieve one or more goals based on the individual abilities, values, preferences, and beliefs [13,14,15]. DM engages complex psychological and neural processes, including competition between automatic and controlled circuits or cognitive and emotional states, as well as the contention between emotional processes [16]. From an anatomical viewpoint, the prefrontal cortex (PFC) is one of the key structures involved in DM [17]. DM appears to depend on the integrity of frontal networks; in particular, the thalamus, the amygdala, and the basal ganglia work in close relationship with other regions. Specifically, the orbitofrontal cortex (OFC) and limbic pathways are directed toward reward and affectivity-based decisions, the dorsolateral prefrontal cortex (DLPFC) is specialized in integrating multiple sources of information, and the anterior cingulate cortex (ACC) is important in sorting among competing options and outcome processing [13,18]. Patients with OFC lesions may suffer from two types of deficits that affect decision making. First, individuals fail to shift stimulus–response contingencies and suffer from impaired reward processing, with consequent difficulties in altering their decisions about stimulus, despite an associated negative outcome [13,18,19,20,21]. Second, patients may be deficient in tasks requiring empathy or theory of mind, failing to process and recognize the emotions of other and manifesting impaired judgment in social contexts [13,18,22,23]. The DLPFC is implied in the retrieval of stored information [24,25] and in the monitoring of working memory [26,27]. This represents an important cognitive requirement in DM, enabling the maintenance of focus on goal hierarchies, monitoring the status of competing options, and possibly storing affective information to attribute and assess options [13,18]. This area is involved during presentation of moral dilemmas, supporting the essential role of the DLPFC in moral reasoning and the process of integrating emotional information into moral judgments [28]. The ACC may regulate autonomic and emotional reactions to events [29] and plays a role in the modulation of other prefrontal regions, such as the OFC and DLPFC [13,18]. This structure participates in performance optimization and evaluation by using previous reward experiences to guide choices; it is recruited for highly ambiguous choices, such as in situations in which conflicting options are presented with a high likelihood of error [13,18,30,31].

However, the PFC is not only responsible for the processes described above; it most frequently activated during episodes of chronic pain [32,33], and it is employed to integrate pain information with other inputs (including memories, mood, and spatial awareness, among others). The result of this combination is used to control pain at the peripheral level through the modulation of nociceptive stimuli [34,35]. However, overlapping of brain networks can limit access to some cognitive resources in patients with chronic pain (CP) because they are engaged in pain-related mechanisms [11]. Cognitive difficulties were identified as a priority by both patients and physicians in surveys investigating the subjective aspects considered particularly relevant in fibromyalgia [36]. This evidence, in addition to impaired cognitive performances reported in fibromyalgia patients compared to healthy subjects, supports the idea that cognitive difficulties are self-reported but also an objective problem with respect to the skills of patients [37]. As indicated in the Outcome Measures in Rheumatology Clinical Trials (OMERACT 9) [38], self-reports of cognitive functions are relevant to capture changes in FM, in addition to performance-based measures. The OMERACT 9 document discusses the need for a more complete understanding of cognitive dysfunction and appropriate measures to detect it, with the purpose of improving research on the topic. The case definition of FM has changed over time, with an increasing recognition of the importance of cognitive problems [39,40] that were not considered in the 1990 American College of Rheumatology (ACR) classification criteria [3]. Cognitive difficulties contribute to disabilities affecting FM patients and can sometimes be more disturbing than widespread pain, changing patients’ lives, sometimes dramatically so [5]. The importance of our systematic review lies in shedding light on a cognitive skill that is often undervalued in the scientific landscape but that is essential in everyday life [15,41] and can be impaired in FM patients. The first evidence of DM impairment in FM subjects (compared to healthy controls) was revealed in a famous task assessing learning performance with respect to rewards and punishments under conditions of risk, ambiguity, and reversing contingencies [20,42,43].

Despite the evidence supporting the relevance of DM skills [15,41], few studies have examined DM in FM sufferers; to the best of our knowledge, ours is the first systematic review on this topic. As mentioned above, the importance of cognitive aspects for the diagnosis of FM has not always been considered [3,39,40], which may help to explain the inclusion of only nine articles in the present review according to the eligibility criteria. Our work was conducted with the aim of identifying articles that evaluated DM performance in people with FM and to highlight the main assessment tools used. An interesting aspect is related to the type of DM analyzed in papers, which is limited to DM under risk or uncertainty, excluding other important areas of DM (e.g., social DM) that could be relevant for a comprehensive understanding of the construct. Therefore, our systematic review not only has the merit of providing an overview of the literature on the theme but also highlights possible gaps to be explored in future research. We suggest that there may be a link between DM and FM that is worth investigating with future research to clarify if, when, and how such difficulties impact the lives of patients.

The identification of impairment is a first step in the process of developing appropriate strategies that can be combined with existing techniques to treat FM, improving the quality of care for FM patients.

## 2. Methods

This systematic review was conducted according to the PRISMA statement [44,45]. This review and its main data collection procedures were preregistered with the Open Science Framework Registries (https://osf.io/bemwk, https://osf.io/rwt79) (Supplementary Materials).

### Research Strategies

An extensive systematic analysis of international literature was conducted without imposing any temporal limitations until 6 September 2022 (date of data extraction) using Scopus, PubMed, Web of Science, Psychology and Behavioral Sciences Collection, Medline Complete, and APA PsycInfo databases. The query strategy included specific terms in combination with Boolean operators for each database consulted. The following words were searched within titles and abstracts: “fibromyalgia”, “decision making”, “emotional function*”, and “cognitive function*”. Owing to differences in the query settings of the databases consulted, for the search carried out on Web of Science, Psychology and Behavioral Sciences Collection, Medline Complete, and APA PsycInfo, we used a two-step process to screen titles and abstracts separately. After conducting a comprehensive search to collect documents containing the selected words in titles or abstracts, we merged papers in one Excel sheet, and any duplicate publications were removed. We filtered these publications selecting only English-language papers and original articles. We then screened the content of titles and abstracts, excluding studies according to the eligibility criteria. Then, the same screening procedure was carried out for full texts. No limitations were implemented with respect to age, gender, or ethnicity.

For the current systematic review, we considered only original articles available in English language and full-text formats, including studies that included FM patients in accordance with international diagnostic criteria for fibromyalgia and with at least one cognitive DM task performed. We selected studies in which authors aimed to analyze DM, including some of its components (e.g., strategic choices taken or formulation and implementation of a plan). The selected papers reported on the use of instruments created specifically for the assessment DM, as well as tools for the assessment of executive function (EF), but only in the specific cases in which authors explicitly mentioned their use in consideration of DM. The dataset was manually reviewed by two authors (A.F. and D.D.), and possible inconsistencies were discussed to reach a final agreement. Any disagreements were resolved through a discussion with a supervisor (author L.T.). Therefore, through a process of interjudge agreement, duplicate publications were removed to include only original papers written in English. A careful reading of titles and abstracts allowed the reviewers to distinguish between relevant articles those not relevant to the scope of the review (e.g., research works or dissertations on medical DM or DM studies non directly regarding patients), and a further selection process was conducted by reading the full texts. Studies that appeared to be unrelated to the review scope or not reporting essential data were excluded (i.e., did not include data regarding DM instruments or performance or concerning participants without a fibromyalgia diagnosis).

Two authors (C.L. and A.F.) independently assessed the quality of the included studies using a version of the Critical Appraisal Skills Programme for the evaluation of case control studies (CASP, 2018 [46]). We selected this checklist because it was appositely developed to control the type of study design implemented in all articles that satisfied the eligibility criteria. The original version of the CASP checklist consisted of 11 questions (with item 6 is divided into 2 parts) structured to systematically guide thinking about study quality. Examples were provided for each item on the checklist to facilitate understanding of the question. A favorable rating was provided if the quality of the topic under analysis was positively regarded. The possible responses admitted were “yes”, “no”, and “can’t tell”. The checklist does not suggest a scoring system, but attributed points to the qualitative responses to obtain a quantitative score. When the quality of the required aspects was evident, one point was assigned, whereas a score of 0 was attributed when the quality was unclear or when the aspects were not considered. The obtained scores for each question were summed, divided by 11 (total number of questions), and transformed into a percentage value by multiplying by 100. We planned to include only articles with quality assessment scores of at least 75%. All 9 articles fulfilled the standard and were included and discussed in this systematic review.

## 3. Results

**Figure 1:**
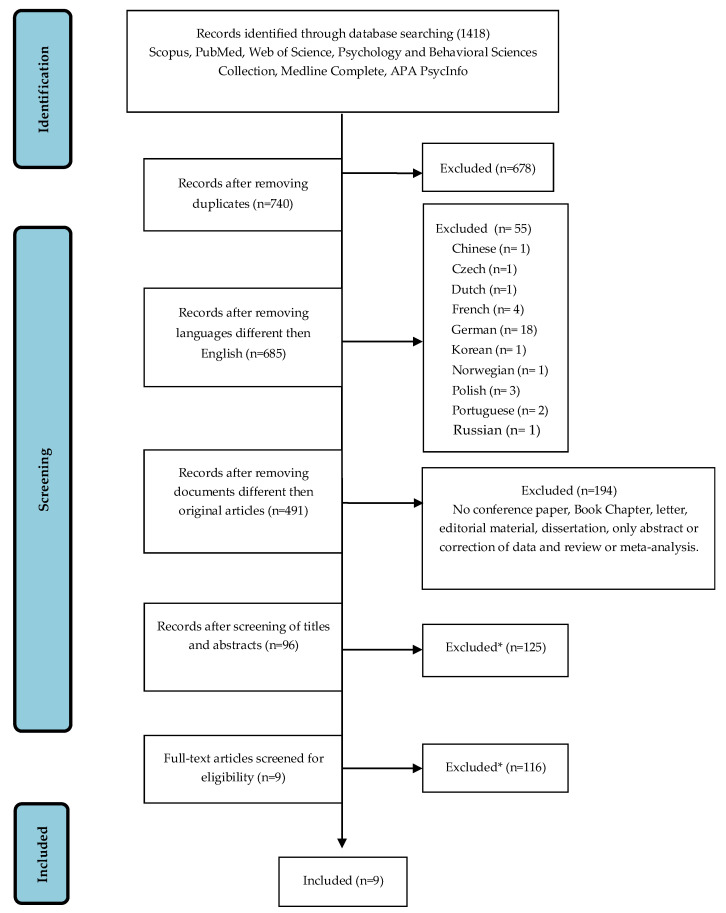
PRISMA flow diagram of the selection process. * Studies not complying with the eligibility criteria were excluded.

In four of the six databases consulted (Web of Science, Psychology and Behavioral Sciences Collection, Medline Complete and APA PsycInfo), owing to their search settings, we used a two-step process to screen titles and abstracts separately; we then excluded duplicates generated within databases. Initially, we compared the various articles from the consulted databases, identifying 740 papers from a corpus of 1418 after elimination of duplicates. References of included papers were screened to include additional articles in this review, but no additional records were added (*n* = 0). Documents any language other than English were excluded (*n* = 55), and only original articles were selected, excluding reviews, meta-analyses, conference papers, book chapters, letters, editorial materials, dissertations, and documents with only an abstract or correction of data (*n* = 194). Reading of the titles and abstracts resulted in the exclusion of irrelevant studies (*n* = 395), and a further selection process was performed by reading the full texts (*n* = 87). Studies that appeared to be unrelated or did not report essential data were excluded (i.e., studies not reporting on DM instruments or performance and studies on participants without a fibromyalgia diagnosis). Ultimately, a total of nine studies were included according to eligibility criteria. The supervisor confirmed that there were no violations of established criteria for article exclusion or inclusion in this systematic review. Figure 1 shows a schematic representation of data sources and the selection process.

### 3.1. Results of the Selected Studies

#### 3.1.1. Demographic Data

The nine studies selected were conducted from 2009 [43] to 2020 [47]. The sample included in this systematic review comprised patients with fibromyalgia (for a critical review, see [48]), with samples principally comprising women, with a total of 615 individuals, of whom 323 were included in clinical groups and 292 in control groups. The characteristics of the participants in the eligible studies are shown in Table 1. Three studies did not report the mean and standard deviation of the level of education of the study sample [47,49,50], and one study did not report the gender of participants [51].

#### 3.1.2. FM Assessment

In most studies, participants with fibromyalgia been diagnosed prior to their recruitment [43,47,49,50,51,52,55]; the disease onset, when specified, is reported in Table 2. Only one study [54] included subjects with generic chronic pain, nine of whom were indicated as having fibromyalgia according to criteria of the American College of Rheumatology Criteria for the Classification of Fibromyalgia [3]. In all cases presented in this review, an assessment of pain or its impact on the individual’s abilities or life was performed (see Table 2). Four studies [49,51,52,54] adopted the visual analogue scale to measure the level of pain experienced by patients on a continuum from “none” to an “extreme amount of pain” [56]. A similar instrument, the numeric rating scale, was employed in two studies [47,50] to provide a measure of pain on a scale from 0 to 10, where 0 represents “no pain” and 10 represents “the worst pain possible” [57]. In three studies [53,54,55], a version of the McGill Pain Questionnaire [58] was adopted, comprising three major classes of words (sensory, affective, and evaluative) used by patients to refer to their subjective pain experience. The individual pain threshold was measured in two of the studies included in this review [47,49,50,54]. Two studies [43,55] employed a version of the West Haven–Yale Multidimensional Pain Inventory [59], providing a brief but comprehensive assessment of the subjective experience of pain, including subjective, behavioral, and psychophysiological components. To measure the components of health status believed to be most affected in persons with FM, in one study [52], the Fibromyalgia Impact Questionnaire [60] was adopted, and in another study [52], participants’ quality of life was tested with the 36-item short-form survey [61]. A further study [53] highlighted the importance of sleep quality with respect to health status through the use of the Oviedo Sleep Questionnaire [62]. Finally, one study [53] employed the fatigue severity scale, measuring the severity of fatigue in different situations during the past week on a scale ranging from 1 to 7 [63].

#### 3.1.3. Cognitive/Emotional Assessment

All studies assessed one or more cognitive functions using standardized battery subscales [64,65,66] or other clinical instruments (see Table 3). In addition to cognitive evaluation, five studies [51,52,53,54,55] also assessed the emotional state of patients [67,68,69]. In one study [53], psychiatric disorders was evaluated [70], whereas personality measures [71] were assessed in another study [43]. Only study [52] included the administration of a screening tool for cognitive abilities [72]. Memory performance was assessed in several studies included in this review. In particular, semantic memory was evaluated in some studies [47,49,50,53] with different instruments [73,74,75]. Short-term memory [76] was assessed in one study [53], and spatial memory was evaluated [65,66,77] in a total of four studies [47,49,50,53]. Attention ratings were considered in two studies [47,50] to obtain measures of reaction time [65] and in another two papers [49,54] to evaluate processing speed, attention, and cognitive flexibility [78]. The Stroop test was used in three studies [51,53,54] to assess the ease with which a person can maintain a goal in mind and suppress a habitual response in favor of a less familiar response [79]. In another study [53], the Stroop effect was explored in a non-verbal mode [80]. Information about cognitive flexibility, rule detection/abstraction, and distraction when monitoring several sources of information [81] was investigated in three studies [43,53,55]. In another study [53], clinical information regarding non-verbal capacity for fluid and divergent thinking, ability to shift cognitive set, planning strategies, and executive ability to coordinate this process were obtained [82]. Finally, in only one study [53], the authors assessed possible malingering [83].

#### 3.1.4. DM Assessment

All studies included in this systematic review had to analyze choice or DM performance to satisfy the inclusion criteria (see Table 3). In three articles, one or more tasks derived from the behavioral assessment of the dysexecutive syndrome were used (BADS [84]). Specifically, the key search test was applied in one of the research works (ZST [53]), whereas the Zoo Map Test was used in two studies (ZMT [49,53]). In the KST, participants had to demonstrate how they would search for a set of lost keys in a field, whereas in the ZMT, subjects had to plan a route to visit locations in a zoo. The ZMT consists of two parts; in the first little part, information is provided to help generate an appropriate plan, and in the second part, participants must follow an externally imposed strategy. In addition, Muñoz Ladrón de Guevara and colleagues (2018) [53] administered the Revised Strategy Application Test [85] as a measure of strategic planning and self-regulation. All these tests, although designed to assess the subject’s ability to formulate and implement a plan, also include a decision-making component. The importance of strategic choices made by decision makers was stressed within the scope of this review. Five studies [43,51,53,55,86] assessed participants’ decision-making behavior in under risk through the Iowa Gambling Task (IGT), which was created to assess real-world decision making in a laboratory setting [87]. For this task, individuals received money and were told to maximize profit during trials by selecting cards from one of four decks. With each draw, decks A and B yielded a major profit relative to decks C and D; however, this was also applied to losses, i.e., selection of cards from decks A and B was more disadvantageous and risky than selections from the other two decks [42]. In one study [51] the Conditional Associative Learning Task [88] was implemented to assess the acquisition of arbitrary associations between targets and colors and to evaluate the ability to discriminate between past correct and incorrect responses and to use this information to guide response selection. This task was used in addition to the IGT to examine the cost that chronic pain may impose on executive control among patients with fibromyalgia. In another case [86], participants performed the Two-Choice Task in addition to the IGT to estimate impulsive choice and DM, evaluating the tendency to choose a small reward over a larger delayed reward [89]. In two studies, the Cambridge Gambling Task was used (CGT; [47,50]) to assess DM and risk taking outside of a learning context. In the CGT, a row of ten boxes, some red and some blue, was presented at the top of a screen. The ratio of red to blue boxes varied between stages, but there was always one box containing a yellow token. Participants had to choose the color of the box in which they thought the token was hidden [65]. Finally, in one study [54], the Game of Dice Task was used to evaluate DM under risk by asking participants to bet on the results of a dice roll. Participants received feedback only after rollout as to whether they gained or lost money [90].

## 4. Discussion

The aim of this systematic review is to determine the link between decision making and fibromyalgia, examining studies that assessed performance on tasks with decision-related features in patients with a fibromyalgia diagnosis. The state of the art showed results on the link between DM and FM that should be cautiously interpreted. Difficulties in decision making in FM patients were considered to be related to symptomatic manifestations of the syndrome and mostly attributed to the simultaneous presence of chronic pain. A hypothesis highlighted among articles included in this review [49] reported an association between nociceptive sensitization and cognitive performance impairments in FM. This assumption revealed that the hyperalgesia characterizing the disorder is congruent with the central pain sensitization hypothesis of FM [91,92,93]. Exaggerated pain processing in FM implies increased demands on central-nervous resources, reducing those available for cognition [49,94]. In another study [54] in which no significant differences in neuropsychological functions were found compared to healthy subjects, it was hypothesized that an influence of dysregulated attention modulation or other dysfunction of the reward/aversion circuit is plausible to explain DM difficulties. The involvement of dysfunction in the reward/aversion circuitry to explain some cognitive difficulties in FM patients [43,54] is supported by evidence that normal functioning of these mechanisms is important for strategic planning and DM and can be altered under prolonged pain conditions [95,96,97,98]. Patients with CP showed more random behavior choice and a slower or no increase in their learning curve based on past rewards or punishments [95,99,100], suggesting an altered reward functioning associated with pain experience. Articles also indicated a possible impairment of executive functioning and emotional states involved in DM using a task engaging an emotional state, such as the IGT [43,51,53,86]. Not all studies reported deficits in this task [55], but the severity and impact of pain was shown to be mostly correlated with obtained performances rather than the level of anxiety, depression, or medication. Another hypothesis identified in this review suggests that lower cognitive performances could be related to dysregulated attention modulation in FM patients [51,54]. In agreement with previous study results, chronic pain might reduce the availability of limited attentional resources for parallel processing of information other than that related to pain [101,102,103,104]. The attentional components outlined by Posner and Petersen (1990) [105] describe several networks of attentional system, including alerting, orienting, and executive functioning. The alerting network prepares the system to react through a change in the internal state and keeps the cognitive system properly activated. The orienting network selectively shifts attention to a potentially relevant area of the visual field. Finally, the executive component of attention is activated during situations that involve planning, maintaining goal-relevant priorities and avoiding interference, detecting errors, providing a new response, overcoming habitual actions, and making decisions [106,107,108]. DM is closely related to executive functions because it falls under the same umbrella concept, which covers all cognitive processes with the purpose of regulating, controlling, and managing other cognitive processes [109]. Finally, several studies included in this work [43,51,53] related DM difficulties to poor access to bodily signals (i.e., interoceptive sensibility) in patients with FM. Bodily signals might guide DM according to the association between each option and somatic responses derived from effects of action in similar situations encountered in the past [110]. FM patients showed general impairment in somatic information processing [111,112], which may have contributed to the difficulties exhibited in associated with FM. This review was not intended to explain causes of DM difficulties in FM but only to provide an overview of the literature on the topic, highlighting that is worth further investigating this topic in future research. Although DM has been the subject of theoretical insights from different areas of psychology and neuroscience, the focus has mainly been on DM in situations of risk or uncertainty, neglecting other areas [15]. It would be interesting to explore other aspects of DM and to clarify if, when, and how these difficulties impact the lives of FM patients. Research on this topic could offer the opportunity to improve the quality of care for patients with FM because it involves a type of skill that is essential for everyday living. Further studies could contribute to improved understanding of the types of cognitive difficulties associated with FM and help to determine whether they depend on individual sensitivity or are typical of the disease. The identification of impairment is a first step in the process of developing appropriate strategies that can be combined with existing techniques for FM treatment. To the best of our knowledge, this is the first systematic review focusing on this topic. There is considerable potential for further study of the interaction between DM and FM, with both psychological and clinical relevance.

## 5. Limitations

The current systematic review is subject to some limitations. First, the tools used for the evaluation of the decision-making process are limited to DM under risk or uncertainty, excluding other important variables required for a comprehensive understanding of the construct, e.g., social DM through tasks to study the relevant phenomena, such as reciprocal exchange, response to fairness and equity, altruism, and punishment [113]. Investigating other key aspects of DM could contribute to a broader understanding of the possible difficulties faced by patients with fibromyalgia, especially in relation to social and interpersonal aspects of choices. Second, the studies reviewed herein were heterogeneous in nature and did not allow for comparison of the results with quantitative analysis. Therefore, we provided a summary of the existing literature dealing with the relationship between fibromyalgia and decision-making performance. Moreover, owing to the applied inclusion criteria, only original articles published in English were reviewed, so the present review is not wholly reflective of the existing literature, and studies written in languages other than English that discuss the relationship between FM and DM may have been overlooked, limiting the generalizability of the reported results. Finally the present review may be limited due to missing information in documents, as well as the small sample of papers included that fulfilled the inclusion criteria. Further research is recommended to evaluate these aspects and to contribute to the expansion of the existing literature on this topic.

## 6. Conclusions

In our systematic review, through consultation of six online databases, we found nine studies conducted between 2009 and 2020 that assessed performance on tasks with decision-related features in patients with a diagnosis of fibromyalgia. An analysis of the studies that met the inclusion criteria showed that there is a link between decision making and fibromyalgia. These results should be cautiously interpreted due to the scarcity of articles that address the topic, mostly of which focus on narrow aspects of DM.

The hypotheses related to the DM performance of patients with FM reported in the nine included papers were discussed in the present review, but our work was not intended to explain the causes of such difficulties. Instead, we intended to provide an overview of the literature on this question, highlighting that this topic is worth investigating through future research. The limited number of articles obtained may also be due to recent changes in the understanding of the importance of cognitive aspects for the diagnosis of FM, which were not considered in the past. DM is an important cognitive function that falls under the umbrella concept of executive functions, which cover all cognitive processes with the purpose of regulating, controlling, and managing other cognitive processes. Considering the relevance of DM skills in everyday life and considering the difficulties encountered by FM patients, we highlight the need to outline the current state of the art, stimulate discussions, and motivate further research on this topic to deeply understand the link between this clinical condition and cognitive abilities. The present review represents a starting point for future research, highlighting the prevalence of DM assessment under conditions of risk or uncertainty compared to other types of DM that might be relevant for a comprehensive understanding of the construct.

## Figures and Tables

**Table 1:** Demographic details of subjects included in the reviewed studies. The numerical data are expressed as mean ± SD or as percentages (%).

Article	Diagnosis	Gender of Clinical Group	Gender of Control Group	Age of Clinical Group	Age of Control Group	Education Level of Clinical Group	Education Level of Control Group
Dualé et al., 2020 [47]	Fibromyalgia	Women		46 ± 10		-	-
Roman et al., 2018 [52]	Fibromyalgia	W = 15; M = 1	W = 13; M = 2	55.00 ± 2.09	50.27 ± 2.03	12.75 ± 0.95	12.27 ± 1.29
Galvez-Sánchez et al., 2018 [49]	Fibromyalgia	Women		50.33 ± 8.76	47.50 ± 7.60	-	-
Muñoz Ladrón de Guevara et al., 2018 [53]	Fibromyalgia	Women		51.25 ± 8.67	52.94 ± 6.59	9.27 ± 3.52	10.59 ± 3.64
Pickering et al., 2018 [50]	Fibromyalgia	Women		48.0 ± 11.0	44.3 ± 9.3	-	-
Masiliūnas et al., 2017 [54]	Chronic pain, including nine subjects with fibromyalgia	W = 16; M = 13	W = 16; M = 14	59.6 ± 6.0	60.7 ± 7.0	13.0 ± 2.9	13.7 ± 2.6
Cuevas-Toro et al., 2014 [55]	Fibromyalgia	Women		48.60 ± 9.255	47.91 ± 10.814	No studies: 5.9% Primary: 31.8% Secondary: 17.6% Diploma: 29.4% Degree: 15.3%	No studies: 4.7% Primary: 29.4% Secondary: 18.8% Diploma: 30.6% Degree: 16.5%
Walteros et al., 2011 [51]	Fibromyalgia	-	-	49.0 ± 6.7	50.4 ± 4.6	<8 years: 33% 8–12 years: 60% >12 years: 7%	<8 years: 7% 8–12 years: 66% >12 years: 27%
Verdejo-Garcia et al., 2009 [43]	Fibromyalgia	Women		45.86 ± 6.78	44.97 ± 6.70	9.42 ± 4.34	10.08 ± 3.22

Abbreviations: W: women; M: men.

**Table 2:** Time since diagnosis of FM and assessment for related symptoms of disease. The numerical data are expressed as mean ± SD; the range is shown in brackets.

Article	Time Since Diagnosis of FM	Assessment for Related Symptoms of FM
Dualé et al., 2020 [47]	5 years (2–7)	NRS Evaluation of skin temperature sensitivity Evaluation of mechanical sensitivity Evaluation of mechanical allodynia TS
Roman et al., 2018 [52]	Clinical group: 8.56 ± 1.47 Control group: 8.47 ± 1.50	VAS FIQ SF-36
Galvez-Sánchez et al., 2018 [49]	Not specified	VAS
Muñoz Ladrón de Guevara et al., 2018 [53]	Not specified	Spanish version of the MPQ FSS OSQ
Pickering et al., 2018 [50]	Not specified	NRS Evaluation of skin temperature sensitivity Evaluation of mechanical sensitivity Evaluation of mechanical allodynia TS
Masiliūnas et al., 2017 [54]	Not specified	VAS Lithuanian analog of the MPQ TPE
Cuevas-Toro et al., 2014 [55]	4.88 ± 3.6	Spanish adaptation of the WHYMPI MPQ
Walteros et al., 2011 [51]	At least 6 months	VAS
Verdejo-Garcia et al., 2009 [43]	3.42 ± 2.32 (0–9)	WHYMPI

Abbreviations: NRS = numeric rating scale; TS = temporal summation; VAS = visual analogue scale; FIQ = fibromyalgia impact questionnaire; SF-36 = 36-item short-form survey; MPQ = the McGill Pain Questionnaire; FSS = fatigue severity scale; OSQ = Oviedo Sleep Questionnaire; TPE = tender points examination; WHYMPI = West Haven–Yale Multidimensional Pain Inventory.

**Table 3:** Clinical/emotional and cognitive assessment. Standardized batteries from which tests were derived are indicated in parentheses.

Article	Clinical/Emotional Assessment	Cognitive Assessment
Dualé et al., 2020 [47]		SoC (CaNTAB) RTi (CaNTAB) CGT (CaNTAB) GNT (CaNTAB)
Roman et al., 2018 [52]	STAI BDI MMSE Free cortisol examinations	Two-choice task IGT
Galvez-Sánchez et al., 2018 [49]		ZMT (BADS) ROCF TAVEC TMT
Muñoz Ladrón de Guevara et al., 2018 [53]	SCID BDI STAI	Letter–number sequencing (WAIS-III) Arithmetic (WAIS-III) Similarities (WAIS-III) Spatial span (WMS-III) Key search test (BADS) ZMT (BADS) N-back task Verbal fluency RFFT SCWIT FDT WCST IGT Revised Strategy Application Test 15-Item Rey Memory Test
Pickering et al., 2018 [50]		SoC (CaNTAB) RTi (CaNTAB) CGT (CaNTAB) GNT (CaNTAB)
Masiliūnas et al., 2017 [54]	HADS	SCWIT TMT GDT
Cuevas-Toro et al., 2014 [55]	HADS	WCST IGT
Walteros et al., 2011 [51]	BDI STAI	Vocabulary (WAIS) Similarities (WAIS) Comprehension (WAIS) Digit span (WAIS) Block design (WAIS) Picture completion (WAIS) CALT SCWIT IGT
Verdejo-Garcia et al., 2009 [43]	TCI-R	WCST IGT

Abbreviations: CaNTAB = Cambridge Neuropsychological Test Automated Battery; SoC = Stockings of Cambridge; RTi = reaction time; CGT = the Cambridge Gambling Task; GNT = graded naming test; STAI = State–Trait Anxiety Inventory; BDI = Beck Depression Inventory; MMSE = Mini Mental State Examination; IGT = Iowa Gambling Task; BADS = behavioral assessment of the dysexecutive syndrome; ZMT = Zoo Map Test; ROCF = Rey–Osterrieth Complex Figure Test; TAVEC = verbal learning test; SCID = Structured Clinical Interview for DSM-5;TMT = Trail Making Test; WAIS = Wechsler Adult Intelligence Scale; WMS = Wechsler Memory Scale; RFFT = Ruff figural fluency test; HADS = Hospital Anxiety and Depression Scale; SCWIT = Stroop color–word interference test; FDT = Five Digits Test; WCST = Wisconsin CARD SORTING TEST; GDT = Game of Dice Task; CALT = conditional associative learning task; TCI-R = Temperament and Character Inventory–Revised.

## Data Availability

Not applicable.

## Supplementary Materials

Tables with details of articles selected according to the implemented eligibility criteria according to PICOS guidelines, the checklist of the quality control and tables with details regarding the PRISMA selection are available on Open Science Framework Registries (https://osf.io/bemwk).
